# 

**Published:** 1880-12-20

**Authors:** 


### *EDITORIAL NOTICES.*

*Death of Dr. Seguin.*—Dr. Edward Seguin died at his residence in New York City, on October 28th, 1880, aged sixty-nine years. Dr. Seguin's specialty was the treatment of Idiocy and all nervous affections, in which he acquired considerable ability.

*The Hammond Prize.*—A prize of $500 is offered by the American Neurological Association to the author of the best essay on the functions of the Thalamus Opticus in Men. Information regarding the same may be had by addressing Dr. F. T. Miles, of Baltimore, or Dr. J. S. Jewell, of Chicago.

*Trommer Extract of Malt Co.*—The advertisement of the above reliable establishment may be seen on inside front cover of our Journal.

These Malt combinations are excellent in quality and variety, and are too well established to require special notice at our hand.

The Company is liberal and even charatable in its dealings. We are pleased to make mention of a recent donation of a large lot of these preparations to the Dispensary of The Southern Medical College in Atlanta. These goods are warmly appreciated by the Faculty, and will prove a blessing to the indigent sick who so largely attend the Dispensary.

*Withdrawals from College Association.*—The following schools are reported as having withdrawn from The College Association: Bellevue Medical College; College of Physicians, New York; Medical Department University of New York; and Rush Medical College.

*Our Advertising Department.*—This Department of our Journal will meet with special attention the ensuing year. We will endeavor to insert the advertisements of none but first-class establishments, and it will be our aim to promote their interest in every legitimate way. As a rule, those who advertise liberally are live business men and deserve the patronage of the public.

We ask our subscribers to read the advertisements, and to encourage their home Merchants and Druggists to patronize them. An we again remind our readers that the advertisements cost them nothing, as we give the same amount of reading matter each month.

*Reed & Carnrick.*—We invite special attention to Reed & Carnrick's new advertisement in this Journal. Their preparations are highly approved and in very general use. The variety of their Maltine Compounds are such as to cover all indications, and are very convenient to the practitioner.

The house is reliable and well established, and does not need any special mention. See their advertisement. It will be found full and satisfactory upon all points.

### *RECEIPTED.*

[Receipts not acknowledged privately are entered here.]

1880—Drs. A. B. Loving, J. R. Wilson, A. J. Ellis, W. B. Thomason, J. J. Grace, J A. Agnew, W. W. Leak, J. A. Gordon, T. N. Skeen, W. Culbertson, Jno. Elsner, C. Bethune, A. A. Stanley. 1881—J. E. Fripp, G. L. Landes, J. R. Moon, J. A. Holloway, C. C. Hammond, A. P. Harris, J. H. Reynolds, T. S. Fox, Jno. T. Lee, J. H. Wysong, J. S. Carothers.

